# An N6-Methyladenosine-Related Gene Set Variation Score as a Prognostic Tool for Lung Adenocarcinoma

**DOI:** 10.3389/fcell.2021.651575

**Published:** 2021-07-08

**Authors:** Huijuan Zhang, Jing Hu, Aina Liu, Huajun Qu, Fenge Jiang, Congcong Wang, Steven Mo, Ping Sun

**Affiliations:** ^1^Department of Oncology, The Affiliated Yantai Yuhuangding Hospital of Qingdao University, Yantai, China; ^2^The 4th Department of Internal Medical Oncology, Harbin Medical University Cancer Hospital, Harbin, China; ^3^CAS Center for Excellence in Brain Science and Intelligence Technology, Shanghai, China

**Keywords:** lung adenocarcinoma, N6-methyladenosine, tumor mutational burden, tumor-infiltrating lymphocytes, prognostic biomarker

## Abstract

N6-methyladenosine (m6A) is the most prevalent type of RNA modification, and we hypothesized that patterns of m6A-related genes may be useful for estimating risk of lung adenocarcinoma (LUAD). An m6A-related gene set variation score (m6A-GSVS) was generated using RNA-sequencing data from LUAD patients in The Cancer Genome Atlas (TCGA). We investigated the association of m6A-GSVS with stemness, tumor mutational burden (TMB), expression of three immune checkpoints, levels of tumor-infiltrating lymphocytes (TILs), and patient prognosis. We found that m6A-GSVS was higher in LUAD than in healthy lung tissue, and it strongly correlated with stemness and TMB. Activated CD4 + T cells were more numerous in LUAD samples that had higher m6A-GSVS than in those with lower scores. Biological processes and pathways, including “Cell cycle,” “DNA replication,” and “RNA degradation,” were significantly enriched in samples with high scores. Furthermore, m6A-GSVS was an independent prognostic indicator in LUAD. In conclusion, we proposed an m6A-GSVS in LUAD. It is a putative indicator for evaluating the ability to RNA m6A, an independent prognostic indicator and associated with tumor stemness.

## Introduction

There are more than 100 posttranscriptional modifications known in eukaryotic RNAs (Roundtree et al., 2017), of which N6-methyladenosine (m6A) is the most prevalent type (Desrosiers et al., 1974; Lee et al., 2014). First discovered in the 1970s (Desrosiers et al., 1974), m6A and its functions did not begin to be studied in depth until recent years when transcriptome-wide profiling of m6A became possible. Increasing evidence suggests that m6A plays crucial roles in the regulation of gene expression by affecting RNA stability, mRNA degradation, translation, and microRNA maturation (Fu et al., 2014; Alarcon et al., 2015; Meyer et al., 2015). Aberrant m6A modification is associated with a variety of cancers (Panneerdoss et al., 2018), including acute myeloid leukemia (Vu et al., 2017), hepatocellular carcinoma (Ma et al., 2017), glioblastoma (Cui et al., 2017), and lung cancer (Choe et al., 2018). Both elevated and decreased levels of m6A are associated with cancer (Liu et al., 2019).

The m6A modification is catalyzed by the methyltransferase complex, also termed a “writer” (Bokar et al., 1994), while demethylase, also known as the “eraser,” removes m6A (Jia et al., 2011; Mauer et al., 2017). The RNA “reader” protein recognizes m6A and is then activated to perform downstream functions (Wang and He, 2014). Levels of m6A are determined by expression of genes encoding writers, erasers, and readers (He et al., 2019), so we hypothesized that the expression patterns of these three types of m6A-related genes may be useful for assessing risk of diseases related to m6A dysregulation.

Aberrant m6A modification has been associated with lung cancer, the most commonly diagnosed cancer and the leading cause of cancer-related deaths (Torre et al., 2015; Choe et al., 2018). Non-small cell lung cancer (NSCLC) represents 85% of all lung cancers, approximately half of which are lung adenocarcinoma (LUAD). To investigate our hypothesis, single-sample gene set enrichment analysis (ssGSEA) was used to calculate an m6A-related gene set variation score (m6A-GSVS) for LUAD patients in The Cancer Genome Atlas (TCGA). We found that LUAD patients with different m6A-GSVS varied in multiple clinical characteristics and prognosis.

## Materials and Methods

### Data Processing

RNA-sequencing data (displayed as raw counts), mutation annotations (aligned against the GRCh38 reference genome), and clinical data from LUAD patients were downloaded from TCGA^1^ using the “TCGAbiolinks” package (Colaprico et al., 2016) in R version 3.6^2^. Gene IDs were converted based on the annotation file (gencode.v33.annotation.gff3), downloaded from GENCODE^3^. Profiles of mRNA expression from the three datasets GSE3141 (Bild et al., 2006), GSE30219 (Rousseaux et al., 2013), and GSE37745 (Botling et al., 2013), all based on the GPL570 platform, were downloaded from the Gene Expression Omnibus (GEO) database using the “GEOquery” package in R^4^. Batch effects were removed from the three datasets using the *ComBat* function in the “sva” package (Leek et al., 2012). If a gene has multiple probes, the average value of the probes was considered the expression of the corresponding gene. The LUAD samples were extracted from the three datasets to validate the prognostic value of m6A-GSVS. Three m6A regulator gene sets were obtained from a previous study (He et al., 2019), comprising seven writer genes (*METTL3*, *METTL14*, *METTL16*, *RBM15*, *VIRMA*, *WTAP*, and *ZC3H13*), two eraser genes (*ALKBH5* and *FTO*), and 11 reader genes (*EIF3A*, *HNRNPA2B1*, *HNRNPC*, *IGF2BP1*, *IGF2BP2*, *IGF2BP3*, *YTHDC1*, *YTHDC2*, *YTHDF1*, *YTHDF2*, and *YTHDF3*).

### Aberrant Expression and Mutation of m6A-Related Genes in LUAD

To identify differentially expressed m6A-related genes, the expression profiles of genes were compared between LUAD tissues and healthy lung tissues in the LUAD dataset from TCGA using the “DESeq2” package (Love et al., 2014). Results were displayed as an expression heatmap, with significance defined as a *p*-value (adjusted by the false discovery rate) < 0.05. The unregulated m6A-related genes were explored in The Human Protein Atlas (HPA^5^). In addition, mutations in those genes were extracted from mutation annotation format (MAF) files using the *GDCquery_Maf* function in the “TCGAbiolinks” package. Mutation frequencies were visualized as a waterfall plot using the *oncoplot* function in the “TCGAbiolinks” package.

### Gene Set Variation Analysis

According to the currently known m6A theory, we conceptualized an “m6A pathway” that is activated by writers and readers and inhibited by erasers: the writers install m6A, erasers delete it, and readers recognize it. Aberrant expression patterns of genes in the pathway are likely to cause abnormal pathway activity. This is analogous to a previous study (Xiao et al., 2019) in which enrichment scores were separately calculated for the activated gene set and repressed gene set in an oncogenic pathway using the “GSVA” package based on ssGSEA. In that study, the enrichment score of the oncogenic pathway was defined as the enrichment score for the activated gene set minus the score for the repressed gene set. Thus, in the present study, the enrichment score of the “m6A pathway” for a given individual was defined as:

m6A-GSVS = “Writer” enrichment score − “Eraser” enrichment score + “Reader” enrichment score.

Gene set variation analysis (GSVA) (Hanzelmann et al., 2013) was used to generate an enrichment score of gene sets based on high-quality studies (Xiao et al., 2019; Zeng et al., 2020). Enrichment scores for the three types of m6A-related gene sets were estimated for each individual using the *gsva* function in the “GSVA” package (Hanzelmann et al., 2013), based on ssGSEA (Barbie et al., 2009; Abazeed et al., 2013). The parameter “method” was set to “ssgsea,” while the parameter “kcdf” was set to “Poisson.” The latter setting indicates that input data are raw counts. Although differences in the expression of some m6A-related genes were not significant, we retained them in the analysis because previous studies showed them to be important regulators of m6A, in accordance with the principle of the GSEA algorithm (Mootha et al., 2003; Subramanian et al., 2005). The median m6A-GSVS was used as the cutoff to stratify all individuals into groups with low or high m6A-GSVS.

### Association of m6A-GSVS With Clinicopathological Features, Tumor Mutation Burden, Stemness, and Immune Checkpoints

The m6A-GSVSs of individuals with different clinicopathological features were compared and visualized using the “ggpubr” package in R^6^. Several studies have linked m6A-related genes with immune responses to immunotherapy and cancer stem cell in various types of cancers (Zhang et al., 2016, 2020; Yang et al., 2019; Wang et al., 2020). Tumor mutation burden (TMB) and the expression levels of immune checkpoints can predict efficacy of treatment with immune checkpoint inhibitors (Kazandjian et al., 2016; Carbone et al., 2017; Hellmann et al., 2018). Thus, we explored the relationships of m6A-GSVS with TMB, stemness, and expression levels of immune checkpoints.

The TMB of each sample was calculated as in a previous study (Lv et al., 2019). We compared the m6A-GSVSs between individuals with high or low TMB and calculated the Pearson correlation between the two variables. In addition, stemness (Malta et al., 2018) was calculated for each individual using the *TCGAanalyze_Stemness* function in the “TCGAbiolinks” package, then compared across healthy lung tissues and tissues from patients with low or high m6A-GSVS. Pearson correlations were explored between m6A-GSVS and the expression levels of three immune checkpoints: PDCD1 (PD1), CD274 (PDL1), and CTLA4.

### Calculation of Tumor-Infiltrating Lymphocytes

Malignant solid tumor tissues comprise not only tumor cells but also tumor-associated normal cells, such as epithelial, stromal, immune, and vascular cells. These cells are thought to have important roles in tumor growth, invasion, and drug resistance (Pages et al., 2005; Kalluri and Zeisberg, 2006; Hanahan and Weinberg, 2011; Straussman et al., 2012). Based on a previous study (Xiao et al., 2019) that generated cell marker gene sets using CIBERSORT (Newman et al., 2015) and MCPcounter (Becht et al., 2016), we estimated the abundances of 24 types of stromal or immune cells for each LUAD sample using ssGSEA in the “GSVA” package (Hanzelmann et al., 2013). The relative abundances of the 24 types of tumor-infiltrating lymphocytes (TILs) were compared between samples with high or low m6A-GSVS.

### Gene Set Enrichment Analysis

In order to explore the biological characteristics of the high m6A-GSVS group, we performed gene set enrichment analysis (GSEA) (Mootha et al., 2003; Subramanian et al., 2005) using the GSEA JAVA program^7^. Three reference gene sets were used from the Molecular Signatures Database v7.1 (Subramanian et al., 2005; Liberzon et al., 2011, 2015): the Kyoto Encyclopedia of Genes and Genomes (KEGG) gene set (c2.cp.kegg.v7.1.symbols.gmt), biological process gene set (c5.bp.v7.1.symbols.gmt), and hallmark gene set (h.all.v7.1.symbols.gmt).

### Survival Analysis

Kaplan–Meier survival curves were compared between individuals with low or high m6A-GSVS using the log-rank method in the “survival” package^8^ and “survminer” package^9^. We carried out uni- and multivariate Cox proportional hazards analyses of the training set in order to compare the prognostic value of the m6A-GSVS with that of routine clinicopathologic features.

### Statistical Analysis

All analyses were performed using R version 3.6 (see text footnote 2). Differentially expressed genes were identified using the unpaired *t*-test. The Shapiro–Wilk test was used to assess whether the m6A-GSVA score was normally distributed. Intergroup differences in continuous variables were assessed for significance using Wilcoxon, Kruskal–Wallis, or unpaired *t*-tests. All tests were two-sided and considered significant if *p* < 0.05, unless otherwise stated.

## Results

### Multiple m6A-Related Genes Aberrantly Expressed While Few Mutated in LUAD

**FIGURE 1:**
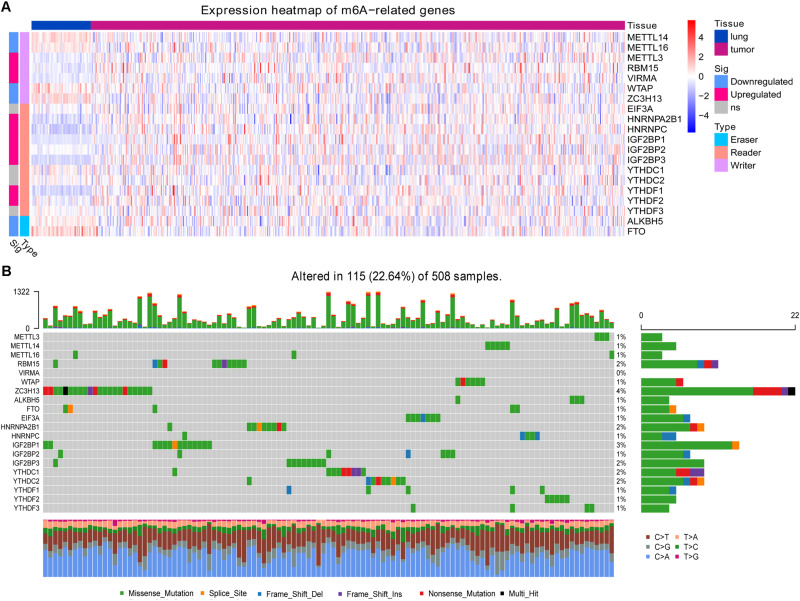
Expression and mutation status of 20 m6A-related genes. **(A)** Differential gene expression between lung adenocarcinoma and healthy lung tissue. **(B)** Mutation status of the genes in lung adenocarcinoma.

In the TCGA-LUAD dataset, we identified multiple m6A-related genes that were aberrantly expressed in LUAD compared with healthy lung tissue (Figure 1A). The writer genes *METTL3*, *RBM15*, and *VIRMA* were upregulated in LUAD, while *METTL14*, *METTL16*, *WTAP*, and *ZC3H13* were downregulated. Most reader genes were upregulated in LUAD (*HNRNPA2B1*, *HNRNPC*, *IGF2BP1*, *IGF2BP2*, *IGF2BP3*, *YTHDF1*, and *YTHDF2*). The two eraser genes, *ALKBH5* and *FTO*, were downregulated in LUAD.

**FIGURE 2:**
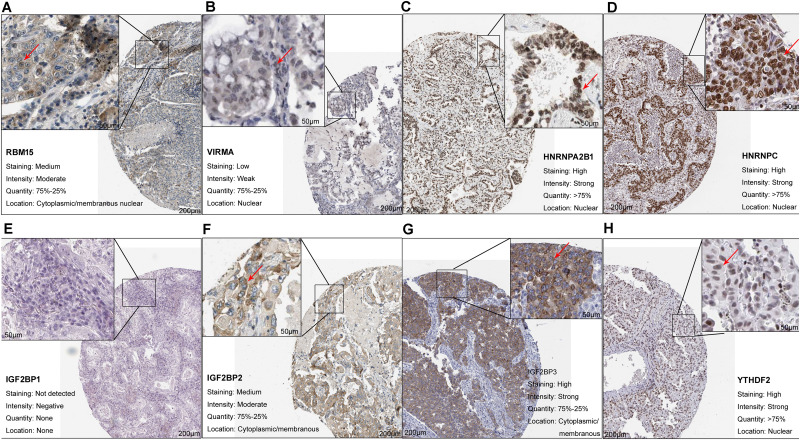
Upregulation of m6A-related genes in lung adenocarcinoma. **(A)** *RBM15* (antibody CAB015201, patient 3003), **(B)** *VIRMA* (antibody HPA031530, patient 1847), **(C)** *HNRNPA2B1* (antibody CAB012403, patient 2222), **(D)** *HNRNPC* (antibody CAB075755, patient 1932), **(E)** *IGF2BP1* (antibody HPA062273, patient 3144), **(F)** *IGF2BP2* (antibody CAB017126, patient 1394), **(G)** *IGF2BP3* (antibody HPA076951, patient 4208), and **(H)** *YTHDF2* (antibody HPA059621, patient 1303).

At least one of these 20 m6A-related genes was mutated in 22.64% of patients with LUAD (Figure 1B). However, each gene was mutated in fewer than 5% of patients. Most of the genes upregulated in LUAD were present in the HPA: *RBM15* (Figure 2A), *VIRMA* (Figure 2B), *HNRNPA2B1* (Figure 2C), *HNRNPC* (Figure 2D), *IGF2BP2* (Figure 2F), *IGF2BP3* (Figure 2G), and *YTHDF2* (Figure 2H). In contrast, the upregulated genes *METTL3* and *YTHDF1* were not included in HPA, while *IGF2BP1* was not detected in LUAD patients (Figure 2E). These results suggest that abnormal m6A levels in LUAD may be driven by aberrant expression, rather than mutation, of m6A-related genes.

### The m6A-GSVS Is Higher in LUAD Tissue Than in Healthy Lung

**FIGURE 3:**
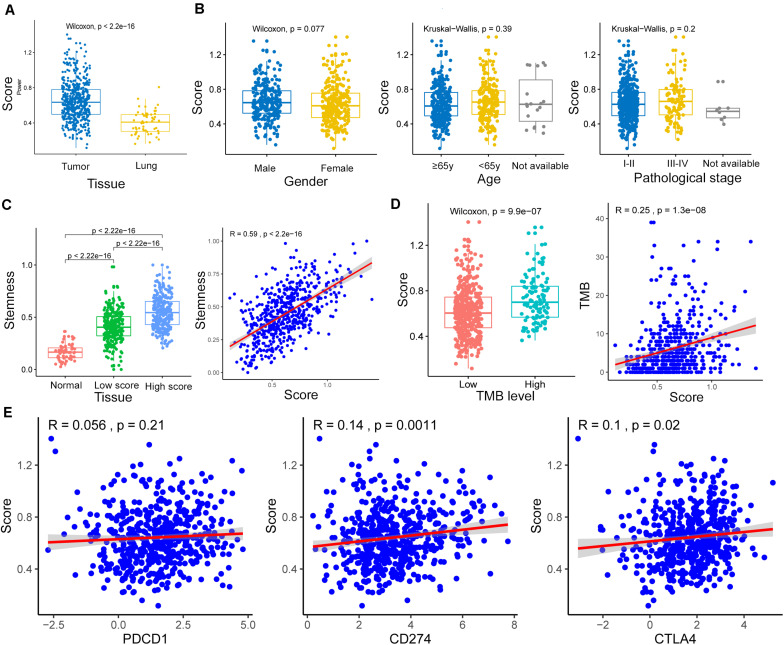
Associations of the m6A-related gene set variation score (m6A-GSVS) with clinicopathological features, stemness, tumor mutational burden (TMB), and expression of three immune checkpoints. **(A)** The m6A-GSVS was higher in lung adenocarcinoma than in healthy lung tissues. **(B)** The m6A-GSVS did not differ significantly with sex, age, or pathological stage. **(C)** The m6A-GSVS correlated strongly with stemness. **(D)** The m6A-GSVS correlated with TMB. **(E)** The m6A-GSVS was associated with the expression of three immune checkpoints.

The m6A-GSVSs of LUAD tissues were significantly higher than those of healthy lung (Figure 3A). This suggests that elevated m6A level may be associated with a higher risk of LUAD. However, m6A-GSVS did not vary significantly with sex, age, or pathological stage among LUAD patients (Figure 3B).

### The m6A-GSVS Correlates Strongly With Stemness and TMB but Weakly With Expression of CD274 and CTLA4

As expected, stemness was significantly higher in LUAD tissue than in healthy lung. We also found that stemness in LUAD patients correlated directly with m6A-GSVS (Pearson *R* = 0.59, *p* < 0.01) (Figure 3C). This suggests that the dedifferentiation of cells into tumors is accompanied by changes in m6A levels. The m6A-GSVS also correlated directly with TMB (Pearson *R* = 0.25, *p* < 0.01; Figure 3D). In contrast, m6A-GSVS showed a weak correlation with the expression of CD274 (Pearson *R* = 0.14, *p* < 0.01) and CTLA4 (Pearson *R* = 0.10, *p* = 0.02) and no correlation with PDCD1 expression (Figure 3E).

### The m6A-GSVS and TILs in the Tumor Immune Microenvironment

**FIGURE 4:**
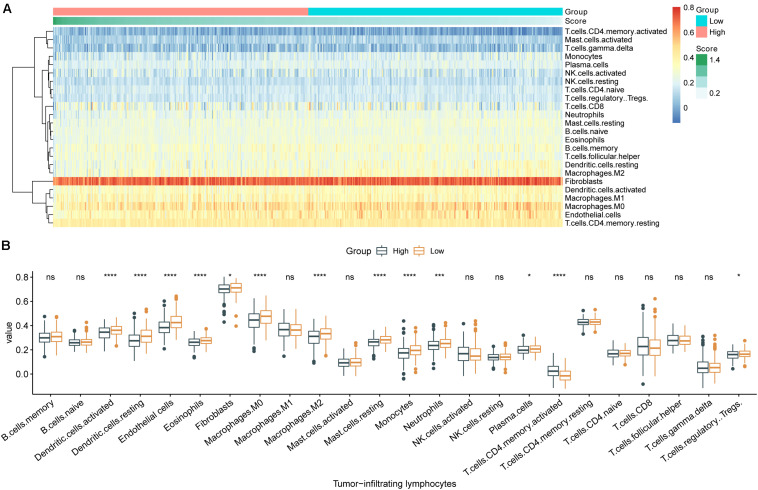
The m6A-related gene set variation score (m6A-GSVS) and the levels of tumor-infiltrating lymphocytes (TILs). **(A)** Heatmap of 24 types of TILs and the m6A-GSVS in each individual. **(B)** Comparison of relative abundances of 24 types of TILs between samples with high or low m6A-GSVS. *p < 0.05, **p < 0.01, ***p < 0.001, and ****p < 0.0001.

The 24 types of TILs in each individual were displayed as a heatmap (Figure 4A). The abundances of nearly all TILs were lower in samples with high m6A-GSVS, including dendritic cells, endothelial cells, eosinophils, fibroblasts, macrophages (M1), resting mast cells, monocytes, neutrophils, plasma cells, and regulatory T cells (Tregs) (Figure 4B). Only activated CD4 T cells were more abundant in samples with high m6A-GSVS than in those with low m6A-GSVS.

### Different m6A-GSVSs in LUAD Samples Are Associated With Differences in Multiple Biological Processes and Pathways

**FIGURE 5:**
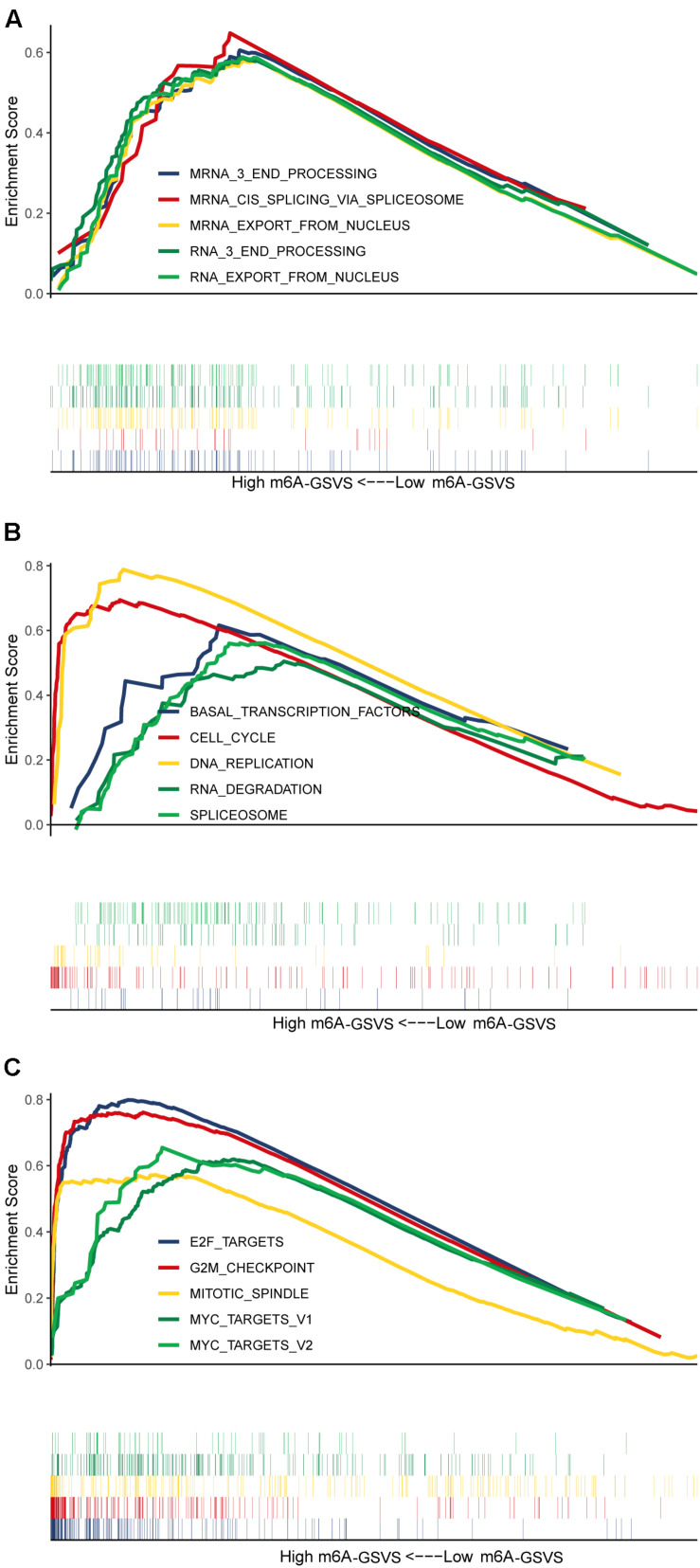
Gene set enrichment analysis. The top five (ranked by false discovery rate) **(A)** biological processes, **(B)** KEGG pathways, and **(C)** hallmark gene sets significantly enriched in samples with high m6A-related gene set variation score (m6A-GSVS).

Based on GSEA, the top five (ranked by false discovery rate) biological processes significantly enriched in samples with high m6A-GSVS were “RNA export from nucleus,” “RNA 3 end processing,” “mRNA 3 end processing,” “mRNA *cis* splicing *via* spliceosome,” and “mRNA export from nucleus” (Figure 5A). The top five KEGG pathways significantly enriched in samples with high m6A-GSVS were “Basal transcription factors,” “Cell cycle,” “DNA replication,” “RNA degradation,” and “Spliceosome” (Figure 5B). The top five hallmark gene sets (Figure 5C) significantly enriched in the high m6A-GSVS samples were “E2F targets,” “G2M checkpoint,” “Mitotic spindle,” “MYC targets v1,” and “MYC targets v2.”

### The m6A-GSVS Is an Independent Prognostic Factor in LUAD

**FIGURE 6:**
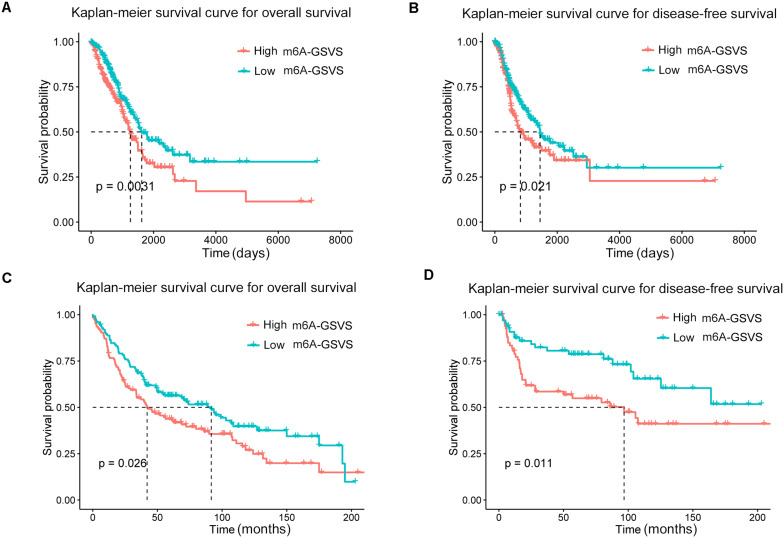
Kaplan–Meier curves for overall survival and disease-free survival of lung adenocarcinoma patients stratified by m6A-related gene set variation score (m6A-GSVS). **(A,B)** Analysis of patient data in the lung adenocarcinoma dataset in The Cancer Genome Atlas. **(C,D)** Analysis of patient data in the GSE3141, GSE30219, and GSE37745 datasets.

In the training set (*N* = 513), univariable Cox analysis showed that higher m6A-GSVS was associated with poor overall survival (OS) [hazard ratio (HR) 2.270, 95% confidence interval (CI) 1.192–4.324, *p* = 0.013]. These results were validated using data from 249 patients with LUAD from the GSE3141, GSE30219, and GSE37745 datasets (HR 5.764, 95% CI 1.549–21.451, *p* = 0.009). Since the m6A-GSVS score showed a skewed distribution, patients were divided into groups with high or low score based on median m6A-GSVS. In the TCGA-LUAD dataset, patients with high m6A-GSVS had worse OS (Figure 6A) and disease-free survival (DFS) (Figure 6B) than those with low m6A-GSVS. Similar results were found for LUAD patients in the GSE3141, GSE30219, and GSE37745 datasets (Figures 6C,D). Multivariable Cox analysis confirmed m6A-GSVS as a prognostic indicator independent of routine clinicopathological characteristics (Table 1).

**TABLE 1:** Univariate and multivariate Cox analyses of the m6A-GSVS and routine clinicopathologic characteristics in patients with lung adenocarcinomas.

Characteristic	Univariate Cox analysis				Multivariate Cox analysis			
	β	HR	HR (95%CI)	*P* value	β	HR	HR (95%CI)	*P* value
**Gender**								
Male	ref.							
Female	0.060	1.061	0.793–1.420	0.689				
Age	0.009	1.009	0.994–1.024	0.259				
**Primary tumor (T)**								
T1–2	ref.							
T3–4	0.861	2.366	1.622–3.451	**0.000**	0.671	1.956	1.281–2.986	**0.002**
Tx	1.284	3.611	0.887–14.693	0.073	1.135	3.112	0.411–23.557	0.272
**Lymph nodes (N)**								
N0	ref.							
N1–3	0.957	2.605	1.938–3.500	**0.000**	0.846	2.331	1.638–3.315	**0.000**
Nx/Not available	0.479	1.615	0.592–4.409	0.995	0.127	1.136	0.279–4.629	0.859
**Metastases (M)**								
M0	ref.							
M1	0.731	2.077	1.214–3.553	**0.008**	0.318	1.374	0.742–2.546	0.312
Mx/Not available	−0.174	0.841	0.587–1.204	0.344	−0.074	0.929	0.647–1.335	0.691
**Pathological stage**								
I-II	ref.							
III-IV	0.962	2.618	1.921–3.567	**0.000**	0.160	1.174	0.757–1.819	0.474
Not available	−0.370	0.691	0.170–2.800	0.604	−0.519	0.595	0.146–2.425	0.469
m6A GSVS	0.820	2.270	1.192–4.324	**0.013**	0.939	2.559	1.310–4.998	**0.006**

*HR, hazard ratio; CI, confidence interval; ref., reference; m6A-GSVS, m6A-related gene set variation score. Bold values *p* represent <0.05.*

## Discussion

Previous studies investigating the aberrant expression of one or two m6A-related genes in cancer (Zhang et al., 2016; Liu et al., 2017; Vu et al., 2017; Hua et al., 2018) have suggested that m6A can regulate the expression of tumor-promoting or tumor-suppressor genes in ways that can promote or inhibit cancer progression (Lin et al., 2016; Choe et al., 2018; He et al., 2018; Wang et al., 2018; Cayir et al., 2019; Zhong et al., 2019). Thus, alterations in the expression of certain m6A-related genes may influence the net effect of m6A alterations on cancer. To explore this idea, the present study looked at multiple validated m6A-related genes as part of an “m6A pathway.” On this basis, we calculated an m6A-GSVS for each sample. We found that most patients with LUAD showed aberrantly expressed m6A-related genes, but only a few patients contained mutations in those genes. This finding is consistent with a previous work (He et al., 2019).

We found that m6A-GSVS was strongly associated with stemness, consistent with a previous report linking m6A level with stem cell fate (Geula et al., 2015). In breast cancer, downregulation of ALKBH5 and m6A may drive cancer stem cell formation (Jaffrey and Kharas, 2017). These observations suggest that m6A may regulate cancer stem cell in certain types of cancer. Further study is needed to clarify the mechanisms linking m6A level and cancer stem cells.

Our analyses showed that m6A-GSVS positively correlated with TMB, and a previous work linked higher TMB with more new transcripts whose protein products act as new antigens (Schumacher and Schreiber, 2015). We infer that tumor cells may increase m6A levels in order to degrade the additional RNAs and thereby avoid attack by immune cells (Fu et al., 2014; Alarcon et al., 2015; Meyer et al., 2015). Consistent with a relationship between m6A level and tumor immunity, m6A modification of mRNA and expression of YTHDF1 in dendritic cells have been associated with antitumor immunity (Han et al., 2019).

As higher TMB is associated with better efficacy of anti-immune checkpoint treatment (Carbone et al., 2017; Hellmann et al., 2018), high m6A level may be associated with response to such treatment in LUAD. For example, m6A level appears to regulate the response to anti-PD1 treatment in melanoma (Yang et al., 2019). However, m6A-GSVS in our study showed weak association with the expression of CD274 and CTLA4, even though the expression of both proteins predicts the efficacy of anti-immune checkpoint treatment (Kazandjian et al., 2016); whether anti-immune checkpoint treatment is more effective for LUAD patients with high m6A-GSVS requires further exploration. Among all the TILs that we analyzed, only activated CD4 + T cells were more numerous in LUAD samples with high m6A-GSVS than in those with low scores. This suggests that higher m6A-GSVS may be associated with greater recruitment of CD4 + T cells, which should be explored in future work.

Consistent with the idea that m6A affects RNA stability and translation (Fu et al., 2014; Alarcon et al., 2015; Meyer et al., 2015), our GSEA indicated that high m6A-GSVS in LUAD was associated with more active nucleic acid metabolism, including “RNA export from nucleus,” “mRNA 3 end processing,” and “mRNA *cis* splicing *via* spliceosome.” High m6A-GSVS was also associated with enrichment in pathways involving “basal transcription factors,” “cell cycle,” “DNA replication,” “RNA degradation,” and the “spliceosome.” These results suggest that the m6A-GSVS may accurately predict m6A activity. In addition, we found that the targets of the transcription factors MYC and E2F were upregulated in LUAD involving high m6A-GSVS, so these two transcription factors may play a crucial role in m6A progression.

We found that the m6A-GSVS was an independent prognostic factor for patients with LUAD: those who had higher m6A-GSVS had worse OS and DFS than those with lower scores. This finding extends studies linking single m6A-related genes to prognosis in various types of cancer (Kwok et al., 2017; Chen et al., 2018; Niu et al., 2019; Liu et al., 2020). Rather than focusing on a single gene, our score evaluates the activity of the “m6A pathway” as a whole.

Our understanding of the mechanism of m6A is still far from complete. The techniques of MeRIP-seq and m6A sequencing have commonly been used to map m6A sites in transcriptomes (Dominissini et al., 2012), but they cannot always determine the number or positions of such sites because a given peak may contain multiple m6A sites (Grozhik and Jaffrey, 2018). Although our present study provided a new insight on m6A RNA modification, it has several limitations. First, the included m6A-related genes are based on previous studies. More such genes may be discovered in future studies, so the m6A-GSVS should be validated and improved accordingly. Second, the m6A-GSVS was designed to estimate the activity of m6A in an individual with LUAD, not in a single RNA molecule. Whether the m6A-GSVS can represent the real level of m6A modifications in LUAD samples requires further study. Third, it is not clear whether m6A-GSVS contributes to prognosis in LUAD or it serves simply as a prognostic marker. Fourth, the lack of data on microsatellite instability in LUAD in TCGA prevented us from exploring its correlation with the m6A-GSVA score, which should be examined given that such instability effectively predicts response to treatment with immune checkpoint inhibitors (Le et al., 2017). Fifth, whether anti-immune checkpoint treatment is more effective for LUAD patients with high m6A-GSVS still requires exploration.

## Conclusion

We propose the m6A-GSVS as an index of m6A activity in LUAD. This score appears to be associated with tumor stemness and may be useful for predicting prognosis.

## Data Availability Statement

Publicly available datasets were analyzed in this study. This data can be found here: https://portal.gdc.cancer.gov/; https://www.ncbi.nlm.nih.gov/geo/.

## Author Contributions

PS and SM designed the study. HZ, JH, AL, and HQ performed the experiment. HZ and JH collected the data and prepared the manuscript draft. HJ and HQ performed the statistical analysis. All of the authors approved the final manuscript.

## Conflict of Interest

The authors declare that the research was conducted in the absence of any commercial or financial relationships that could be construed as a potential conflict of interest.

## Footnotes

1 https://portal.gdc.cancer.gov/

2 https://www.r-project.org

3 https://www.gencodegenes.org/human/

4 https://bioconductor.org/packages/GEOquery/

5 https://www.proteinatlas.org/

6 https://CRAN.R-project.org/package=ggpubr

7 http://www.broadinstitute.org/gsea

8 https://CRAN.R-project.org/package=survival

9 https://CRAN.R-project.org/package=survminer
